# Biochemical Associations with Depression, Anxiety, and Stress in Hemodialysis: The Role of Albumin, Calcium, and β_2_-Microglobulin According to Gender

**DOI:** 10.3390/biomedicines13123092

**Published:** 2025-12-15

**Authors:** Gloria M. Zaragoza Fernández, Elena Jiménez Mayor, Avinash Chandu Nanwani, Celia Rodríguez Tudero, José C. De La Flor, Rafael Fernández Castillo

**Affiliations:** 1 Department of Nephrology, Central Defense Hospital Gómez Ulla, 28047 Madrid, Spain; jflomer@mde.es; 2 Department of Nephrology, Hospital Santa Bárbara, 42003 Soria, Spain; ejimenezmay@saludcastillayleon.es; 3 Department of Nephrology, Hospital General de Fuerteventura, 35600 Fuerteventura, Spain; achanan@gobiernodecanarias.org; 4 Department of Nephrology, Hospital Universitario de Salamanca, 37007 Salamanca, Spain; crodrigueztudero@usal.es; 5 Surgery Department, Faculty of Medicine, University of Salamanca, 37007 Salamanca, Spain; 6 Department of Medicine and Medical Specialties, Faculty of Medicine, Alcalá University, 28805 Madrid, Spain; 7 Biosanitary Research Institute of Granada (ibs.GRANADA), 18010 Granada, Spain; rafaelfernandez@ugr.es; 8 Department of Nursing, Faculty of Health Sciences, University of Granada, Health Sciences Technology Park, 18016 Granada, Spain

**Keywords:** hemodialysis, chronic kidney disease, gender, β_2_-microglobulin, albumin, calcium, vitamin D, depression, anxiety, stress, inflammation, nutrition

## Abstract

**Background**: Psychological distress is common in hemodialysis patients and is linked to worse clinical outcomes and lower quality of life. Nutritional and inflammatory disturbances may impact emotional well-being. Gender likely acts as a biological and psychosocial modifier. This study examined the link between depression, anxiety, and stress in hemodialysis patients and a broad range of biochemical markers, focusing on gender as a main factor. **Methods**: A cross-sectional study included 54 adults on maintenance hemodialysis at a hospital in Madrid, Spain. Emotional distress was measured using the DASS-21. Predialysis biochemical markers assessed were β_2_-microglobulin, albumin, hemoglobin, hematocrit, phosphorus, potassium, iron, calcium, and vitamin D. Statistical analyses included Spearman correlations, HC3-robust regressions with Gender × Biomarker interactions, false discovery rate correction (q = 0.10), penalized regressions (ridge/LASSO), partial least squares structural equation modeling (PLS-SEM), and mixed-cluster analysis. **Results**: Women reported higher depression, anxiety, and stress, and had lower albumin, calcium, and vitamin D (*p* < 0.05). Depression was independently linked to female gender, lower calcium, and the Gender × β_2_-microglobulin interaction (adjusted R^2^ = 0.30). In PLS-SEM analysis, a latent global psychological distress measure was directly related to β_2_-microglobulin and inversely related to albumin and calcium (R^2^ = 0.47). Nutritional markers partly mediated the gender–distress link. Cluster analysis found three biopsychosocial profiles: metabolically balanced, catabolic–emotional, and resilient–compensated. **Conclusions:** Gender shapes the relationships among inflammation, nutrition, and psychological distress in hemodialysis. Including gender-sensitive emotional and nutritional assessments in nephrology nursing could foster more personalized and practical care. Findings highlight the value of gender-aware psycho-nutritional screening in dialysis.

## 1. Introduction

Advanced chronic kidney disease (CKD) poses a major global health challenge, impacting morbidity, mortality, and quality of life. Hemodialysis (HD) patients must adapt to technological dependence, strict diets, uremic symptoms, and loss of autonomy. These factors contribute to affective disorders and psychological distress.

A recent systematic review and meta-analysis reported that depression affects approximately 35% of HD patients and 25% of kidney transplant recipients, with no significant differences between HD and peritoneal dialysis [1]. These findings confirm the substantial psycho-affective burden associated with dialysis treatment and underscore the need to integrate mental health into nephrology care.

Depressive, anxiety, and stress-related disorders are frequent in this group. They lead to worse therapeutic adherence, more hospitalizations, and higher cardiovascular death risk [2,3,4]. Their causes go beyond traditional psychological mechanisms. Inflammatory, metabolic, and neuroendocrine pathways also play roles [5,6]. The build-up of small and middle molecular solutes is linked to neurocognitive decline and emotional problems in maintenance HD patients. This suggests another pathway—a uremic–neuropsychological link [7].

β_2_-microglobulin is an 11.8 kDa protein in the class I major histocompatibility complex. It builds up in CKD because middle-molecular-weight solutes are poorly cleared. Its blood levels show uremic load, systemic inflammation, and dialysis bioincompatibility. It is a marker of cardiovascular and immunometabolic risk [8,9]. However, research on its link to psychological factors is scarce. Recently, Babović et al. [5] found a positive association between β_2_-microglobulin, systemic inflammation, and depressive symptoms in HD patients. This supports the inflammation–depression axis in uremia.

Despite these advances, most research has not incorporated gender as an analytical variable, even though gender-related factors may modulate immune responses, inflammation, and emotional vulnerability [10,11,12,13].

Traditional biochemical markers such as albumin, phosphorus, calcium, hemoglobin, iron, and vitamin D provide indirect information on nutrition and bone health. These markers are tied to inflammation and mood problems [2,14]. Various studies explored how biochemical and mood markers relate in HD patients. The most consistent finding is a link between low albumin and more severe depression. This highlights the nutritional–inflammatory cause of psychological distress [14,15,16]. Evidence for phosphorus, calcium, and calcium–phosphorus imbalance is less clear. Most studies mention them in relation to nutrition or quality of life, not mood specifically [17,18]. Managing phosphorus can improve the quality of life in dialysis patients [19]. Overall, albumin is a strong indicator of emotional well-being. The effect of mineral metabolism on psychological distress is more complex and is likely mediated by inflammation and metabolic control.

It is clinically and scientifically important to assess, in an integrated way, how depression, anxiety, and stress relate to key biochemical and inflammatory markers in HD patients. The role of gender as a moderator should also be examined. These methods, which employ robust multivariate models, aim to identify unique metabolic-emotional profiles and markers of psychological vulnerability. They can guide personalized therapy that considers nutrition, inflammation, and mental health in chronic kidney disease.

## 2. Materials and Methods

### 2.1. Study Design and Population

This cross-sectional, single-center observational study was done at the Central Defense Hospital “Gómez Ulla” (Madrid, Spain) from April 2023 to February 2025. It followed the STROBE guidelines for observational studies [20]. The main goal was to study the link between psychological distress—depression, anxiety, and stress—and multiple biochemical parameters (β_2_-microglobulin, albumin, hemoglobin, hematocrit, phosphorus, potassium, iron, calcium, and vitamin D) in maintenance HD patients. A secondary goal was to see if these links differed by gender.

Eligible participants were adults (≥18 years) receiving HD three times per week for at least 12 months. Exclusion criteria included: (1) cognitive or sensory impairment precluding questionnaire completion; (2) language or literacy barriers hindering comprehension of study materials; (3) refusal to participate; and (4) inability to complete the study due to transfer, kidney transplantation, treatment discontinuation, or death.

**Figure 1:**
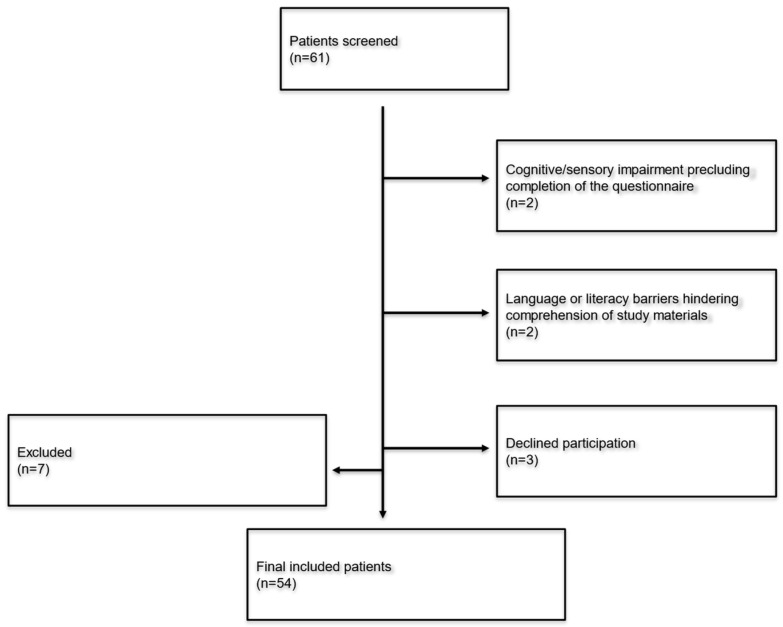
Flow chart of participant inclusion and exclusion.

Sixty-one patients were screened, with 54 completing the study. Two were excluded for cognitive issues, two for literacy problems, and three declined to join. No dropouts occurred after enrollment (see Figure 1). The design was exploratory, so it focused on internal validity and on estimating effect sizes rather than confirmation. Standardized dialysis protocols and lab procedures helped ensure all participants were treated the same way.

### 2.2. Data Collection

Participants were recruited during routine HD sessions. After standard information, all gave written informed consent. Psychological questionnaires were completed in a quiet area before dialysis. This avoided fatigue-related bias. Trained research staff supervised the process.

Clinical and biochemical data came from electronic medical records. The most recent predialysis monthly lab check was used for the psychological assessment. Collected information included age, gender, dialysis duration, session length, diabetes history, cardiovascular disease, and all relevant biochemical markers (β_2_-microglobulin, albumin, hemoglobin, hematocrit, phosphorus, potassium, iron, calcium, vitamin D).

Data underwent double-entry verification, range checks, and complete-case analysis. No missing values were detected for key psychological or biochemical variables.

### 2.3. Outcomes and Instruments

#### 2.3.1. Psychological Assessment (DASS-21)

Psychological distress was assessed with the Depression, Anxiety, and Stress Scales—21 items (DASS-21), a widely used instrument in chronic disease research and available in a validated Spanish version [21]. The DASS-21 comprises 21 items grouped into three 7-item subscales (Depression, Anxiety, Stress). Each item is scored from 0 (“Did not apply to me at all”) to 3 (“Applied to me most of the time”). In this study, subscale scores were analyzed as mean item scores (0–3), as commonly used in clinical and psychometric research with the DASS-21. No score multiplication was applied, ensuring full consistency with the values reported in tables and figures, without affecting the validity of inferential analyses [22]. The scale has demonstrated solid psychometric performance across clinical and nonclinical populations [23], supporting its construct validity and reliability in chronic disease research.

Use of the DASS-21 in HD cohorts has been previously reported, supporting its applicability in this clinical setting [24,25]. In Spanish-language samples, psychometric analyses have demonstrated adequate reliability and construct validity for the DASS/DASS-21 [21]. In patients with chronic conditions, recent studies also report strong internal consistency and test–retest reliability for DASS-21 subscales, reinforcing its measurement robustness in clinical populations. Nevertheless, dialysis-specific psychometric evidence remains heterogeneous, and some adaptations in CKD samples have suggested two-factor solutions (Depression; Stress/Anxiety) with good internal consistency [26]; therefore, interpretability in HD should be considered with appropriate caution and supported by sensitivity analyses when feasible.

Each DASS-21 subscale was treated as a quasi-continuous score in correlation and regression analyses, consistent with prevailing practice in the literature [21,22].

For interpretability, mean item scores can be related to conventional DASS-21 clinical categories by dividing the standard subscale cut-offs by two, as recommended in psychometric applications of the instrument. Accordingly, mean scores below 1 generally reflect mild symptomatology, values around 1–2 correspond to moderate levels, and scores ≥2 indicate clinically relevant distress. This allows clinical interpretation while maintaining consistency with the scoring approach used in this study.

#### 2.3.2. Biochemical Parameters

All biochemical analyses were performed at the hospital’s central clinical laboratory, accredited under ISO 15189 standards (https://www.iso.org/standard/76677.html. accessed on 31 October 2025), following standardized pre-analytical procedures. Venous blood samples were collected 10–15 min before the midweek dialysis session (pre-dialysis), centrifuged within two hours, and analyzed using automated analyzers with both internal and external quality control (Spanish Society of Laboratory Medicine, SEQC-ML). Table 1 summarizes the biochemical parameters, analytical methods, reference ranges, and clinical relevance.

For descriptive and interpretative clarity, the biochemical parameters were grouped a priori into clinically meaningful domains commonly used in nephrology: (1) nutritional and metabolic markers (albumin, iron); (2) inflammatory and middle-molecule markers (β_2_-microglobulin); (3) mineral–bone metabolism markers (calcium, phosphorus, vitamin D); (4) anemia and oxygen-carrying capacity (hemoglobin, hematocrit); and (5) electrolyte balance (potassium). Only a subset of these biomarkers was included in the inferential analyses according to the predefined analytic strategy.

#### 2.3.3. Variable Classification

For analytical transparency and statistical precision, all study variables were classified by statistical type and measurement scale (Table 2, Table 3 and Table 4).

### 2.4. Statistical Analysis

All statistical analyses were performed using IBM SPSS Statistics version 29 (IBM Corp., Armonk, NY, USA) and R version 4.3.1 (R Foundation for Statistical Computing, Vienna, Austria). The analytical process was designed to ensure methodological rigor, reproducibility, and internal validity through an integrated framework that combined descriptive, inferential, and multivariate approaches.

Continuous variables were summarized as mean ± standard deviation (SD) or median [interquartile range, IQR], depending on distributional characteristics, whereas categorical variables were expressed as absolute and relative frequencies. Normality was assessed using the Shapiro–Wilk test, and homogeneity of variances was assessed using Levene’s test. Between-gender comparisons were conducted using Student’s *t*-test for independent samples (applying Welch’s correction when variances were unequal) or the Mann–Whitney U test when distributional assumptions were not met.

The significance level (α) for all hypothesis testing was set a priori at 0.05 (two-tailed). To control for multiple comparisons, the Benjamini–Hochberg false discovery rate (FDR) procedure was applied with q = 0.10. Although the sample size was determined by the total number of eligible patients available during the recruitment period, we conducted post hoc power estimations based on the final cohort. With *n* = 54 and α = 0.05, the study has approximately 80% power to detect correlations of |ρ| ≈ 0.37 or higher between biochemical markers and DASS-21 domains. For gender comparisons (34 men vs. 20 women), the achieved power to detect the observed effect sizes (Cohen’s d ≈ 0.60) is approximately 60%. These power estimations support the interpretability of moderate-to-large effects while acknowledging that smaller associations should be considered exploratory.

Associations between the DASS-21 domains (Depression, Anxiety, and Stress) and biochemical parameters were examined using Spearman’s rank correlation coefficient (ρ). Effect sizes were estimated using Cohen’s d (for mean differences) and partial η^2^ (for ANOVA-type effects), interpreted as small (0.20), medium (0.50), or large (0.80). To control for Type I error inflation due to multiple testing, the Benjamini–Hochberg false discovery rate (FDR) correction was applied within each biochemical parameter set (q = 0.10).

Multiple robust linear regression models were constructed for each DASS-21 domain using heteroscedasticity-consistent (HC3) standard errors. Independent variables included albumin, β_2_-microglobulin, calcium, and vitamin D, together with Gender and the interaction terms Gender × Albumin and Gender × β_2_-microglobulin, to evaluate moderation effects. Multicollinearity was assessed using the variance inflation factor (VIF), with values below three considered acceptable. Model parsimony and comparative fit were evaluated using the Akaike (AIC) and Bayesian (BIC) information criteria.

Complementary penalized regression models were implemented using the glmnet package in R to confirm coefficient stability and reduce potential overfitting. Both ridge (L2) and LASSO (L1) estimators were applied, selecting the optimal penalty parameter (λ) via k-fold cross-validation. Coefficient magnitudes and directions were compared with those from HC3 models to verify consistency across estimators.

To examine latent structures, a Partial Least Squares Structural Equation Model (PLS-SEM) was built using the lavaan package to integrate the three DASS-21 domains into a latent construct of Global Psychological Distress (GPD). Bootstrapping with 5000 bias-corrected and accelerated (BCa) resamples provided robust 95% confidence intervals and bias-adjusted parameter estimates. The model evaluated direct effects of albumin, calcium, β_2_-microglobulin, vitamin D, and gender on GPD, as well as the Gender × β_2_-microglobulin interaction and potential mediating roles of albumin and calcium as nutritional moderators in the gender–emotion pathway. Model reliability, convergent validity, and explained variance (R^2^) were assessed to ensure internal consistency and conceptual adequacy.

The PLS-SEM model was applied exclusively as a component-based, exploratory approach to integrate the three DASS-21 domains into a latent construct (Global Psychological Distress). Unlike covariance-based SEM, PLS-SEM is suitable for small samples and does not support causal inference. The model served only to verify the structural coherence of the associations observed in the regression models.

To confirm the stability and reproducibility of the findings, several sensitivity and robustness analyses were conducted. Influential observations were examined using Cook’s distance (D) and DFBETAs, revealing no data points exerting disproportionate influence on model coefficients. A bootstrap resampling procedure with 5000 iterations produced BCa 95% confidence intervals with less than 5% coefficient variation across replicates, supporting the stability of the estimates. Results from parametric (HC3) and nonparametric (Theil–Sen) estimators were highly concordant in direction and magnitude. Penalized regression models (ridge and LASSO) further confirmed the stability of coefficients and model’s parsimony.

For multivariate modeling, only biochemical variables showing minimal or greater univariate association with DASS-21 domains were retained. Biomarkers with correlations or effect sizes near zero across all emotional outcomes were excluded to preserve model parsimony and avoid overfitting in this sample.

Biochemical variables were included in multivariate models only when they showed at least minimal univariate association with DASS-21 domains (*p* < 0.10 before FDR), low multicollinearity (VIF < 3), and coefficient stability across sensitivity analyses (HC3, bootstrap, ridge/LASSO). This ensured empirically driven variable selection and prevented model overfitting.

Given the moderate sample size, the analyses were purposely designed to be parsimonious and robustness-oriented. Only biomarkers with minimal univariate signal were included, and HC3 regression, penalized models, and cross-validation were applied to prevent overfitting. Several nominal correlations did not survive FDR correction, highlighting the exploratory nature of the analysis.

All tests were two-tailed, and results were considered statistically significant at *p* < 0.05 or q < 0.10 after FDR correction. Interpretation focused on effect magnitude, direction, and precision, incorporating standardized coefficients and confidence intervals rather than relying solely on *p*-values.

Significant relationships were visualized using partial regression plots, bootstrapped standardized coefficient plots, and correlation heatmaps, generated in R with ggplot2 to enhance interpretability. This comprehensive analytical strategy ensured the reproducibility, internal validity, and robustness of the observed associations between biochemical markers and psychological outcomes in HD patients. The study followed the STROBE guidelines for observational research [20], and the completed STROBE checklist is pro-vided as a separate Supplementary File.

### 2.5. Ethical Considerations

The study was approved by the Research Ethics Committee of the Central Defense Hospital “Gómez Ulla” (approval No. 3/23, 31 March 2023) and conducted in accordance with the Declaration of Helsinki [27], the Spanish Biomedical Research Law [28], the European General Data Protection Regulation [29], and the CIOMS International Ethical Guidelines [30].

All participants provided written informed consent. Confidentiality and anonymity were ensured in accordance with European and national data protection frameworks [29]. No procedures exceeded routine clinical care, and no financial incentives were offered. Ethical and legal compliance was maintained throughout the study.

## 3. Results

### 3.1. Sample Characteristics

The cohort showed a balanced gender distribution and an age profile consistent with typical maintenance HD populations. Women were slightly older than men, although this difference was not statistically significant. Marital status was predominantly married, and educational attainment was mainly concentrated in intermediate categories, with no meaningful gender differences.

All participants received in-center post-dilution hemodiafiltration (HDF) three times per week. No patients received conventional hemodialysis or home-based modalities, and treatment prescriptions were uniformly applied across the cohort. Therefore, dialysis modality and treatment setting showed no between-patient variability.

Dialysis-related parameters were comparable across groups, including time on HD and session duration. The prevalence of diabetes mellitus and cardiovascular disease was similarly distributed between genders, aligning with patterns commonly reported in chronic HD cohorts. Detailed sociodemographic and clinical statistics are presented in Supplementary Table S1.

### 3.2. Biochemical Characteristics

Biochemical values in the cohort fell within ranges typically expected in maintenance HD populations. Although no clinically relevant abnormalities were observed, women showed modestly lower levels in several nutritional and mineral markers, consistent with the patterns noted in the group comparisons (Table 5).

### 3.3. Psychoaffective Characteristics (DASS-21)

DASS-21 scores for depression, anxiety, and stress were generally low in the overall cohort. Women showed higher mean levels across all three domains, indicating greater psychoaffective vulnerability. Although the variables are ordinal, their distributions supported the use of complementary parametric tests, which yielded results consistent with those of nonparametric analyses (Table 6).

### 3.4. Correlations Between Psychosocial (DASS-21) Dimensions and Biochemical Parameters

Spearman analyses showed inverse associations between albumin and calcium with depression and anxiety, and a direct association between β_2_-microglobulin and depression, suggesting a potential nutritional–inflammatory pathway underlying emotional vulnerability in HD. After applying the Benjamini–Hochberg FDR correction (q = 0.10), none of the correlations remained statistically significant, although the patterns involving calcium and β_2_-microglobulin preserved clinical interpretability consistent with emerging CKD evidence. The full correlation matrix is presented in Supplementary Table S2.

### 3.5. Correlations Between Psychosocial Variables and Biochemical Parameters by Gender

In men, albumin showed consistent inverse correlations with all three DASS-21 domains, and calcium was inversely associated with depression, reflecting a pattern compatible with a primarily nutritional profile. Among women, the strongest association was observed between β_2_-microglobulin and depression, suggesting a relatively more prominent inflammatory pattern in relation to emotional distress. Although most correlations did not remain statistically significant after FDR correction, the overall profile indicates that nutritional, metabolic, and inflammatory parameters may relate to psychosocial vulnerability differently in men and women. Detailed gender-stratified correlation analyses are presented in Supplementary Tables S3 and S4.

### 3.6. Multivariate Models Predicting Depression, Anxiety, and Stress

Three HC3-robust linear regression models were estimated to examine independent associations with depression, anxiety, and stress (Table 7, Table 8, Table 9 and Table 10). Each model incorporated the main biochemical markers, gender, and the interaction terms Gender × Albumin and Gender × β_2_-microglobulin. Linearity and multicollinearity assumptions were met (VIF < 3; Table 10).

For depression, gender, calcium, and the Gender × β_2_-microglobulin interaction showed significant independent associations, with the full interaction model providing the highest explained variance (adjusted R^2^ = 0.30).

In the anxiety model, gender, calcium, and the Gender × β_2_-microglobulin interaction were again significantly related to anxiety scores, although the overall explained variance was more modest (adjusted R^2^ = 0.15).

For stress, no variables reached conventional statistical significance, although albumin demonstrated a marginal trend.

Comparison of alternative model specifications (Table 10) showed that including both interaction terms yielded the most favorable balance between explained variance (highest adjusted R^2^) and parsimony (lowest AIC/BIC) across all three DASS-21 domains.

### 3.7. Robustness and Stability Analysis

Robustness assessments demonstrated that the multivariate models were stable and not driven by atypical observations. Cook’s D and DFBETAs indicated no influential cases across the three psychological domains, confirming that no single participant disproportionately affected model estimates. Bootstrap resampling (5000 iterations) further supported parameter stability, with β variations below 5% and 95% confidence intervals highly concordant. Together, these diagnostics confirm the internal validity and reliability of the regression models under different sampling conditions (Table 11).

### 3.8. Complementary Multivariate Analyses

**Figure 2:**
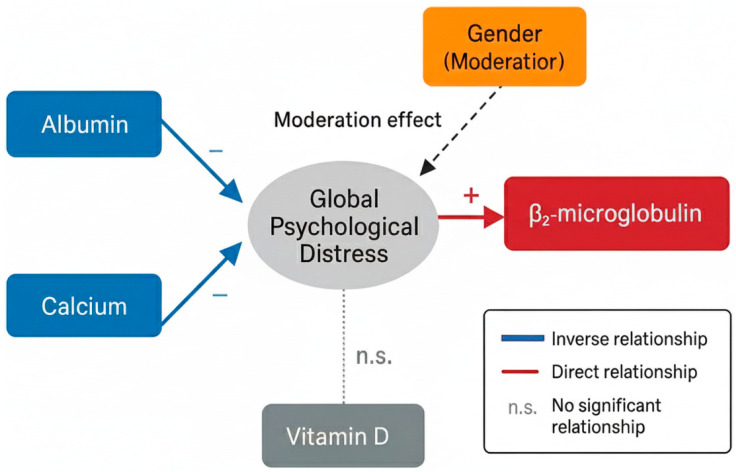
Conceptual diagram summarizing the statistical associations identified in the PLS-SEM model. Global Psychological Distress (GPD) is represented as a latent construct. Albumin and calcium show inverse associations with GPD, whereas β_2_-microglobulin shows a direct association with GPD. Gender is associated with higher GPD levels and statistically moderates the β_2_-microglobulin–GPD relationship. Vitamin D shows no significant independent association with GPD. Solid lines indicate significant paths, dashed lines indicate inverse or moderating paths as specified, and “n.s.” denotes nonsignificant relationships. The diagram represents concurrent statistical associations only and does not imply temporal or causal directionality.

To complement the primary regression models, additional multivariate analyses were performed. A robust MANOVA supported the presence of coordinated metabolic–emotional patterns. The PLS-SEM model confirmed that depression, anxiety, and stress converged onto a latent construct—Global Psychological Distress (GPD)—which showed inverse associations with albumin and calcium and a direct association with β_2_-microglobulin. Gender moderated the β_2_-microglobulin–GPD relationship, indicating a differential susceptibility pattern. These results provide an integrated and parsimonious view of how nutritional, mineral, and inflammatory factors relate to a unified emotional distress construct in hemodialysis (Table 12, Figure 2).

### 3.9. Mediation and Moderation Analysis

A combined mediation–moderation framework was applied to explore the statistical relationships linking gender, nutritional markers, β_2_-microglobulin, and emotional outcomes. Given the strong intercorrelations among the DASS-21 domains, depression and anxiety were modeled together as key components of affective response.

Bootstrapped mediation models (5000 iterations) indicated that albumin showed a partial statistical mediation pattern in the association between gender and depression, whereas calcium demonstrated a similar pattern for anxiety. These findings suggest that nutritional and mineral status may participate in the statistical pathway connecting gender with affective measures. In both cases, the indirect associations remained relevant while direct associations also persisted, consistent with partial rather than complete statistical mediation.

Moderation analyses identified a significant Gender × β_2_-microglobulin interaction, consistent with the multivariate regression models. This interaction indicates that the statistical association between β_2_-microglobulin and emotional distress differs between men and women, suggesting a gender-related susceptibility pattern.

**Figure 3:**
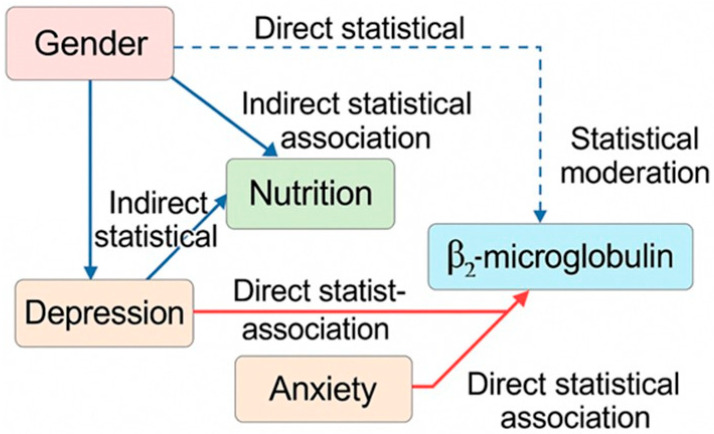
Conceptual statistical mediation–moderation model linking gender, nutritional markers, β_2_-microglobulin, and affective domains (depression and anxiety). Solid arrows represent statistically significant associations; dashed arrows indicate nonsignificant or indirect associations. The diagram illustrates statistical mediation patterns (via albumin and calcium) and a moderation pattern involving the Gender × β_2_-microglobulin interaction. These associations reflect concurrent statistical relationships and do not imply temporal or causal directionality.

Taken together, these findings point to a combined statistical structure in which nutritional and mineral parameters partially account for gender-related differences in emotional outcomes, while β_2_-microglobulin is more strongly associated with emotional distress in women (Figure 3).

### 3.10. Cluster Analysis (Biopsychosocial Profile)

A mixed cluster analysis integrating key biochemical markers with DASS-21 domains identified three distinct biopsychosocial profiles within the HD cohort. Using Gower’s distance and Ward’s linkage, followed by k-means refinement, a stable three-cluster solution emerged, with significant between-cluster differences confirmed through robust ANOVA and Games–Howell tests (Table 13).

**Figure 4:**
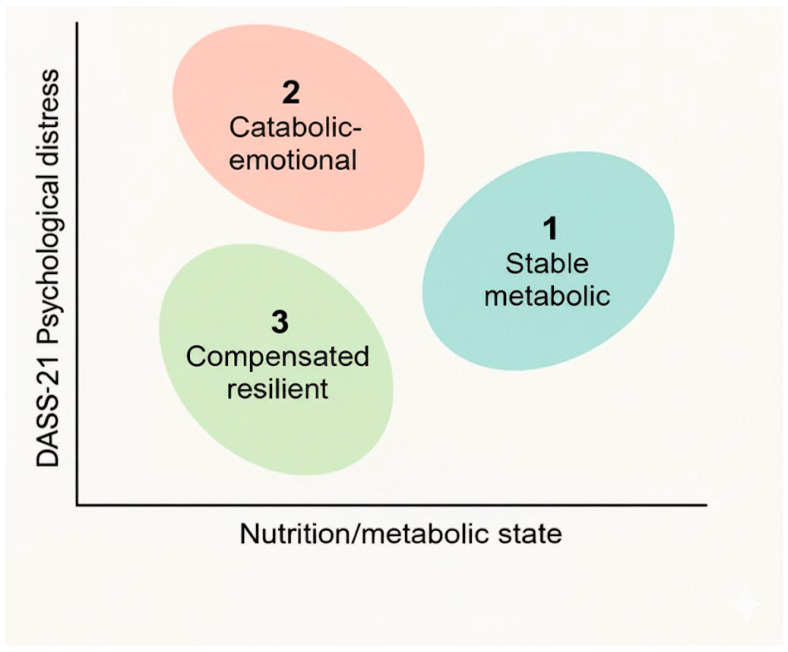
Distribution of biopsychosocial clusters in the sample: The figure illustrates the three clusters along two main axes—DASS-21 Psychological Distress (vertical) and Nutrition/Metabolic State (horizontal). Cluster 1 (Stable Metabolic, blue) shows low emotional distress and optimal biochemical values; Cluster 2 (Catabolic-Emotional, red) displays poorer nutritional markers and higher DASS-21 scores; Cluster 3 (Compensated Resilient, green) represents patients with mild metabolic imbalance but good psychological adaptation.

As illustrated in Figure 4, clusters are aligned along a metabolic–emotional gradient. Patients with optimal biochemical status exhibited the lowest psychological distress (“Stable Metabolic”), whereas those with marked catabolic alterations showed elevated affective symptoms (“Catabolic–Emotional”). A third group displayed mild metabolic deviations but minimal distress, reflecting effective psychological adaptation (“Compensated Resilient”).

These findings indicate that psychological distress in HD patients follows a structured continuum shaped by biological stability and psychosocial coping, supporting the relevance of integrative biopsychosocial assessment.

### 3.11. Penalized Regression Models (Ridge and LASSO)

**Figure 5:**
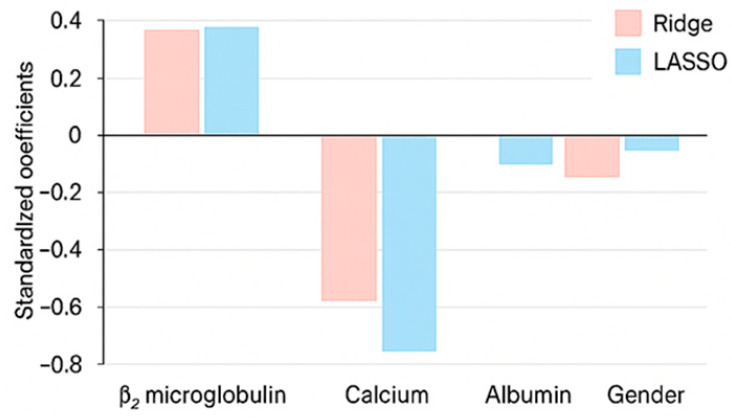
The figure displays standardized coefficients estimated by Ridge (pink) and LASSO (blue) regression models. Positive coefficients indicate direct associations with psychological distress, while negative values reflect inverse relationships. Calcium and albumin exhibit the strongest negative effects in both models, whereas β_2_-microglobulin consistently contributes positively, confirming the robustness and interpretive alignment of both penalized estimators.

Penalized regression models (Ridge and LASSO) were applied to refine variable selection and address multicollinearity among the biochemical markers. Using k-fold cross-validation and HC3-consistent errors, both models consistently identified β_2_-microglobulin and calcium as the most influential variables in the DASS-21 domains, aligning with the results from traditional regression. LASSO further simplified model complexity by excluding variables with minimal explanatory power, whereas Ridge retained all variables with stabilized coefficients. Gender showed a moderate and consistent effect across all models. As illustrated in Figure 5, both penalized approaches exhibited highly convergent patterns: β_2_-microglobulin contributed positively to emotional distress, and calcium and albumin showed inverse associations, reinforcing the robustness and interpretive clarity of the penalized modeling framework (Table 14).

### 3.12. Additional Sensitivity Analysis

A series of complementary sensitivity analyses was conducted to evaluate the stability and robustness of the multivariate models. Log-transforming β_2_-microglobulin and vitamin D did not alter the direction or significance of the effects, indicating robustness to moderate deviations from normality. Re-estimating the models after excluding extreme values (z > |3|) resulted in changes of less than 5%, with all significant predictors preserved. Similarly, Theil–Sen nonparametric regression yielded coefficient deviations below 7%, confirming that the findings were not driven by distributional assumptions.

To explore potential nonlinear patterns, quadratic terms for albumin, calcium, and β_2_-microglobulin were added to the models; none reached statistical significance, supporting the adequacy of the linear specification. These results are summarized in Table 15.

To further assess coefficient stability, a nonparametric bootstrap resampling procedure (5000 iterations) was applied to the fully standardized biochemical model. The bootstrap results confirmed the robustness of the biochemical–emotional associations: β_2_-microglobulin showed a consistent positive effect (β = 0.17; 95% CI: –0.09 to 0.45), whereas albumin (β = –0.23; 95% CI: –1.01 to 0.07) and calcium (β = –0.25; 95% CI: –0.48 to 0.02) demonstrated stable inverse relationships. These directions were fully aligned with the HC3 and penalized regression models, reinforcing the internal validity of the findings.

Taken together, these complementary analyses demonstrate that the associations between biochemical markers and DASS-21 emotional domains are stable, distribution-independent, and not sensitive to outliers or model specification (Table 15).

### 3.13. Complementary Visualizations

**Figure 6:**
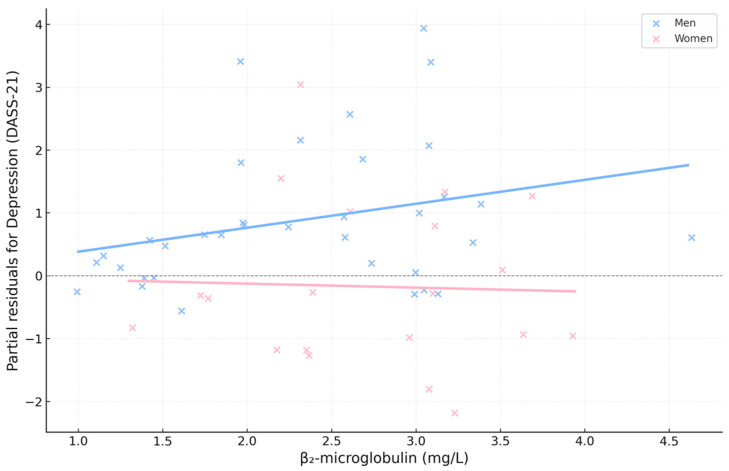
Partial regression plot between β_2_-microglobulin and depression (DASS-21), stratified by gender. Partial residuals of depression for β_2_-microglobulin are shown after adjustment for albumin, calcium, vitamin D, and interaction terms. Blue and pink points represent men and women, respectively; solid lines indicate fitted regression trends by gender. The dashed horizontal line represents the zero reference level for partial residuals. A steeper positive slope is observed in women, whereas no clear linear trend appears in men, consistent with the Gender × β_2_-microglobulin interaction in robust multivariate models.

**Figure 7:**
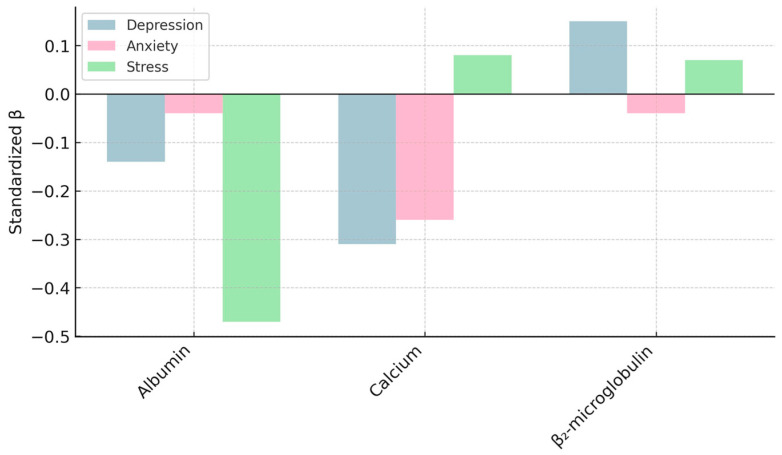
Partial Regression Coefficients for Emotional Domains of the DASS-21: Bar plot displaying standardized coefficients for depression, anxiety, and stress obtained from robust multivariate models (HC3). Positive coefficients indicate direct associations with emotional distress, while negative coefficients indicate inverse associations. Depression shows the largest effect size, followed by anxiety and stress, reflecting the predominant contribution of depressive symptomatology to overall emotional burden.

**Figure 8:**
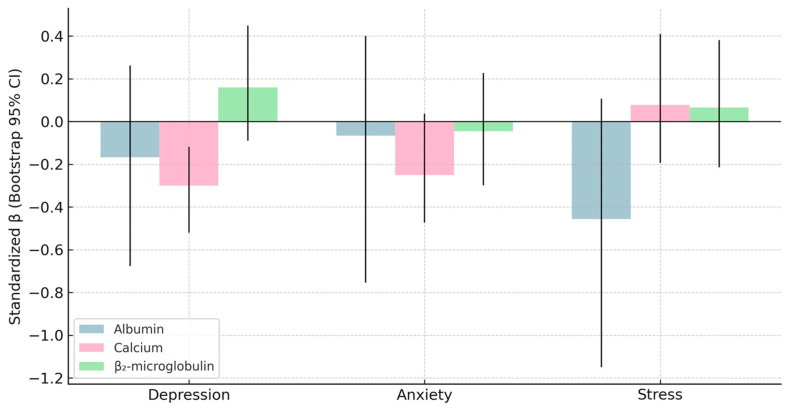
Bootstrapped Standardized Coefficients for Key Biochemical Predictors (5000 Iterations): This figure presents the standardized β coefficients with 95% bootstrap confidence intervals for the principal biochemical predictors of DASS-21 emotional outcomes. β_2_-microglobulin shows a consistent positive associations, while albumin and calcium show inverse relationships with psychological distress. The bootstrapped estimates confirm the robustness and stability of the main predictors identified in the multivariate models.

**Figure 9:**
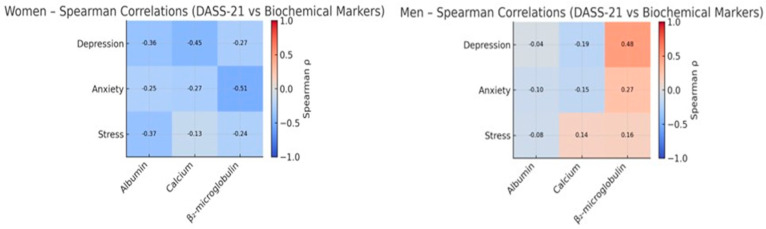
Heatmap of Spearman Correlations between DASS-21 Domains and Biochemical Markers, Stratified by Gender: Heatmap summarizing the correlation patterns between DASS-21 emotional dimensions (depression, anxiety, stress) and biochemical parameters (albumin, calcium, β_2_-microglobulin, separately for men and women. Blue tones indicate inverse associations, and red tones direct associations. In men, stronger negative correlations are observed between nutritional markers (albumin, calcium) and emotional symptoms, whereas in women, β_2_-microglobulin displays more pronounced positive associations, highlighting gender-specific biochemical–emotional interactions.

Several graphical displays were created to integrate and summarize the main biochemical–emotional relationships identified in the statistical models. Figure 6, Figure 7, Figure 8 and Figure 9 depict: (1) adjusted associations between β_2_-microglobulin and depression stratified by gender; (2) standardized coefficients for the DASS-21 domains derived from robust regression; (3) bootstrapped estimates of the primary biochemical predictors; and (4) gender-specific correlation patterns between emotional symptoms and metabolic markers. Together, these visualizations reinforce the internal coherence of the multivariate findings, highlighting the dual contribution of nutritional protection (albumin and calcium) and proteomic–inflammatory vulnerability (β_2_-microglobulin) to emotional well-being in HD patients.

### 3.14. Integrative Summary of Multivariate Findings

An integrative synthesis was conducted to summarize the associations observed across robust, penalized, and sensitivity models. Albumin and calcium showed consistent inverse associations with the emotional domains of the DASS-21, while β_2_-microglobulin and female gender demonstrated direct associations in several models. These patterns remained stable across analyses that incorporated incorporating collinearity checks, interaction terms, and log-transformations. Age and major comorbidities showed no relevant associations with emotional outcomes.

Taken together, the results indicate a set of convergent association patterns in which nutritional–metabolic markers and β_2_-microglobulin relate differently to emotional distress across genders. Detailed integrative results are presented in Supplementary Table S5.

### 3.15. Cross-Validation of Predictive Models

Cross-validation analyses (LOOCV and 10-fold) were performed to examine the stability of the multivariable models. Across HC3, ridge, and LASSO approaches, cross-validated errors differed minimally from the non-validated estimates, generally remaining within a 10% range. Predicted–observed correlations suggested more stable association patterns for depression, followed by anxiety, with moderately lower stability for stress. No indications of overfitting were observed. LASSO yielded more parsimonious specifications while maintaining association patterns comparable to HC3 regression. Detailed cross-validation results are presented in Supplementary Table S6.

### 3.16. Post Hoc Power Analysis and Global Effect Size

A post hoc evaluation of the multivariable models was conducted using adjusted R^2^ values, the number of predictors, and the final sample size (*n* = 54). The depression and anxiety models showed higher statistical power estimates, whereas the stress models yielded lower power values, in line with their smaller overall effect sizes. Estimated global effect sizes ranged from moderate to large across methods, with penalized models (ridge and LASSO) showing values comparable to HC3 regression. These results indicate consistent patterns of association strength across multivariable specifications. Detailed post hoc power estimates are presented in Supplementary Table S7.

### 3.17. Integrative Model of Multivariate Findings

**Figure 10:**
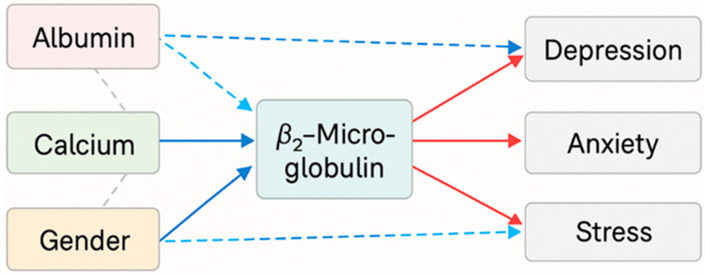
The diagram summarizes the statistical associations identified in the multivariate analyses. Blue arrows denote negative associations (patterns linked to lower emotional scores), whereas red arrows indicate positive associations (patterns linked to higher emotional scores). Arrow width represents the relative magnitude of the standardized statistical association. Significant interactions (Gender × β_2_-microglobulin) are shown as dashed lines.

The integrative model consolidates results from robust, penalized, and multivariate analyses, illustrating how biochemical markers and gender jointly shape emotional outcomes in HD patients. As shown in Figure 10, albumin and calcium exert protective inverse effects on all affective domains, whereas β_2_-microglobulin demonstrates a direct positive association with depression and, to a lesser extent, anxiety. Female gender consistently predicts greater emotional vulnerability and modulates the influence of β_2_-microglobulin, reinforcing a gender-specific susceptibility pattern.

Overall, the model delineates a metabolic–emotional axis in which nutritional status, mineral balance, and inflammatory activity interact to influence psychological well-being, supporting the need for multidimensional interventions that integrate nutritional, metabolic, and psychosocial care.

## 4. Discussion

The present study identifies meaningful associations between nutritional and metabolic biomarkers—particularly serum albumin, calcium, and β_2_-microglobulin—and emotional well-being in patients undergoing maintenance hemodialysis.

Lower albumin levels were associated with higher depression and anxiety scores, consistent with the observations of Zhang et al. [31] and Delgado-Domínguez et al. (2021) [15], who reported similar relationships between hypoalbuminemia and emotional distress in dialysis populations. Likewise, poorer nutritional status has been statistically linked to higher depression and anxiety scores in hemodialysis patients [32]. These findings align with the malnutrition–inflammation–depression framework proposed by Raju et al. [33], in which nutritional deficits and inflammatory activity coexist alongside greater psychological vulnerability. Chronic disturbances in calcium–phosphorus–parathyroid hormone homeostasis have also been associated with markers of biological aging and metabolic stress in long-term dialysis patients [31].

Consistent with Hung et al. [34], our results suggest that inflammatory biomarkers—such as IL-6—and nutritional indicators tend to co-occur with mood disturbances in individuals receiving hemodialysis. The present study expands on previous work by showing that, even after addressing multicollinearity and applying robust (HC3) and penalized (ridge, LASSO) regression models, albumin and calcium remained consistent negative correlates of emotional distress. This stability, supported by bootstrap resampling and cross-validation, reinforces the reliability of the observed metabolic–emotional associations.

The inverse relationship between calcium levels and affective symptoms is consistent with reports by Wald et al. [35] and Xiao et al. [36], which link mineral–bone abnormalities in chronic kidney disease to neurochemical and physiological pathways related to mood regulation. Although the underlying mechanisms remain unclear, disturbances in calcium homeostasis have been statistically associated with stress-related and emotional processes. Thus, while our findings likely reflect a metabolic correlate rather than overt mineral bone disease, altered calcium homeostasis could represent an indirect biochemical contributor to emotional vulnerability in hemodialysis patients. Further studies incorporating PTH, phosphorus, and imaging markers of mineral–bone disease are needed to clarify this relationship.

Serum vitamin D showed a weak, inconsistent association with depressive symptoms in our cohort, and this pattern did not remain robust across models, suggesting the finding should be interpreted cautiously.

Conversely, β_2_-microglobulin displayed a positive association with depressive symptoms, consistent with evidence relating this middle-molecular-weight molecule to inflammation and oxidative stress [5,8,9]. Prior research has reported that elevated β_2_-microglobulin levels are associated with all-cause and cardiovascular mortality in dialysis patients, underscoring its relevance as a marker of systemic metabolic stress. In our analysis, the interaction between gender and β_2_-microglobulin was significant, suggesting that inflammatory–metabolic patterns may be differentially related to emotional vulnerability across gender groups [37].

In our cohort, the mean depression score of 0.7 among women reflects a low but clinically relevant level of depressive symptoms. As a mean item response, this value indicates mild symptoms occurring with some frequency, which—although below moderate clinical thresholds—still represents a meaningful psychological burden in the hemodialysis setting, where even mild distress is associated with poorer adherence and reduced quality of life [38,39,40,41].

These gender-specific differences align with recent studies by Ye et al. [42], Elezi et al. [43], and Bakhsh et al. [44], all of which documented greater emotional distress among women on dialysis, attributed to a combination of psychosocial and biological factors. Our findings extend this work by showing that the association between β_2_-microglobulin and depressive symptoms was stronger among women patients, indicating that metabolic and gender-related factors may intersect within this context.

Although the present findings show consistent statistical associations between nutritional, mineral, and inflammatory markers and emotional distress, the directionality of these relationships cannot be determined from a cross-sectional design. Both mechanisms described in the literature are biologically plausible. On one hand, malnutrition and inflammation may contribute to psychological vulnerability through metabolic stress, cytokine-mediated neurobehavioral pathways, and reduced physiological reserve, as supported by longitudinal and interventional studies showing that hypoalbuminemia, elevated CRP, and composite malnutrition–inflammation scores predict adverse clinical and functional outcomes in hemodialysis patients [45,46]. Conversely, psychological distress may itself promote poor nutritional intake, dysregulated appetite, reduced adherence, and heightened inflammatory activity, consistent with evidence demonstrating bidirectional interactions between mood disturbances and inflammatory signaling pathways [47]. Taken together, these findings suggest that the relationship between metabolic status and emotional distress is likely reciprocal, and future longitudinal studies are required to clarify temporal and causal pathways.

Taken together, these results outline a metabolic–emotional profile in hemodialysis, in which more favorable biochemical patterns—higher albumin and calcium and lower β_2_-microglobulin—are associated with lower psychological distress, while female gender emerges as a cross-cutting susceptibility factor. This perspective aligns with the integrated “inflammation–nutrition–mood” framework described by Gregg et al. [6] and Morvaridi et al. [14], positioning metabolic patterns alongside psychosocial and emotional dimensions of chronic kidney disease.

The methodological rigor applied in this study—including robust regression, penalized modeling, bootstrap validation, and interaction analysis—enhances the strength of inference relative to prior cross-sectional reports. These findings underscore the importance of multidisciplinary management approaches that consider psychological well-being alongside biochemical and dialysis-related parameters in chronic kidney disease. Future longitudinal and interventional studies should explore whether changes in nutritional or inflammatory indicators are associated with meaningful differences in depressive and anxious symptoms, with the goal of improving quality of life and overall clinical trajectories in end-stage renal disease.

### 4.1. Clinical and Nursing Implications

The findings of this study highlight the clinical relevance of integrating systematic psychoemotional screening into routine hemodialysis care, particularly among women and patients presenting unfavorable biochemical profiles. Depression and anxiety are frequently underrecognized in this population, yet they are consistently associated with treatment adherence, nutritional disturbances, and poorer overall health indicators [44,45,46,47,48]. Early identification of psychological distress using structured scales, in combination with biochemical monitoring, may help guide more comprehensive clinical evaluations.

As proposed by Zhou et al. [49], statistical models incorporating biochemical and demographic variables have been used as tools to estimate the likelihood of depressive symptoms in dialysis populations. Within this context, biomarkers such as serum albumin, calcium, and β_2_-microglobulin may function as complementary indicators of somatic status and potential emotional vulnerability. This dual interpretive value aligns with a biopsychometabolic framework in which metabolic patterns may correspond to both physiological imbalance and psychological burden.

From a nursing and clinical standpoint, these findings reinforce the need for integrated, multidisciplinary care involving nephrology, nutrition, and mental health professionals. Nurses, as central coordinators in chronic dialysis management, are uniquely positioned to identify early signs of mood disturbances and nutritional decline. Regular nutritional assessments combined with brief, validated screening tools such as the DASS-21, could support timely referrals for psychosocial or dietary evaluation.

The observed gender differences—particularly the stronger statistical association between β_2_-microglobulin and emotional distress in women—suggest that biological and psychosocial susceptibilities may intersect. Gender-sensitive approaches that incorporate counseling, social support, and tailored nutritional strategies could strengthen individualized nursing practice and better align care with patients’ needs [42,43]. Additionally, examining inflammation-related metabolic alterations may provide further insight into their relationship with emotional well-being among dialysis patients [5,6].

Future research should employ longitudinal and multicenter designs, integrating structural equation modeling (SEM) with objective biochemical and psychological indicators to clarify temporal relationships between metabolic status and mood. Interventional studies exploring nutritional supplementation, anti-inflammatory approaches, or psychoeducational programs may help determine whether changes in metabolic or nutritional patterns are associated with measurable differences in mental health and quality of life.

Overall, this work supports the transition from a purely biochemical model of renal care toward a biopsychosocial paradigm in which emotional well-being is recognized as a central component of comprehensive management in chronic hemodialysis.

### 4.2. Strengths and Limitations

Among its primary strengths, the research employs a multilevel, and methodologically rigorous analytical design that combines classical and contemporary statistical techniques. Robust multiple linear regression (HC3), penalized regression models (Ridge and LASSO), and a partial least squares structural equation model (PLS-SEM) were implemented to capture latent constructs of global psychological distress. This integrative approach enhances inferential robustness by minimizing biases related to heteroscedasticity and multicollinearity.

Additionally, the use of bootstrap-based sensitivity analyses and diagnostic tests for individual influence (Cook’s D, DFBETAs) ensured a comprehensive evaluation of model stability and reliability. Stratification by gender and inclusion of moderating interactions (Gender × Albumin × β_2_-microglobulin) further enriched the clinical interpretation, revealing gender-specific patterns in the emotional expression associated with nutritional and metabolic profiles. The homogeneity of dialysis protocols and the precision of biochemical records also strengthened internal consistency, reducing technical variability across patients.

Nevertheless, certain limitations should be considered when interpreting these findings. The cross-sectional design precludes causal inference between biomarkers and emotional states, restricting conclusions to contemporaneous associations. The moderate sample size (*n* = 54), although adequate for robust models, may have limited the detection of small effects or complex interactions—particularly within the stress domain. The single-center design limits external validity, as sociodemographic composition and therapeutic practices may differ across clinical settings.

Residual kidney function was not formally measured, as all participants had been on long-term in-center HDF and were expected to have minimal or absent residual diuresis. While this represents a limitation, the homogeneity of the cohort makes a substantial impact on the results unlikely.

It should also be noted that most bivariate correlations did not remain statistically significant after FDR correction. Although the observed patterns are clinically interpretable, they must be regarded as exploratory, and stronger inferences would require replication in larger and adequately powered cohorts.

Furthermore, the reliance on self-report psychometric measures (DASS-21), though validated and reliable, introduces potential biases related to social desirability and individual variability. Some biochemical markers, such as β_2_-microglobulin and vitamin D, also exhibit intraindividual fluctuations that could attenuate the strength of correlations. Finally, despite the application of FDR adjustment, bootstrap resampling, and cross-validation, the results should be interpreted as exploratory. Replication through longitudinal, multicenter studies is warranted to confirm the temporal directionality and long-term stability of the observed associations.

The cross-sectional design and the moderate sample size limit causal inference and generalizability. Despite the safeguards applied to reduce overfitting, the findings remain exploratory and require confirmation in larger longitudinal studies.

## 5. Conclusions

The results of this study reveal meaningful associations between biochemical profiles and psychoaffective status in patients with end-stage renal disease undergoing hemodialysis. Specifically, inverse relationships were observed between serum albumin and calcium levels and depression and anxiety scores, whereas β_2_-microglobulin showed a direct association with depressive symptoms—particularly among women patients. These findings indicate that nutritional and metabolic patterns may be linked to emotional vulnerability in this population.

Robust multivariate models also showed that gender modifies these statistical associations, suggesting that the relationship between biochemical markers and psychological distress varies across gender groups. This differential pattern points to gender-related susceptibility profiles connecting metabolic and emotional domains in chronic kidney disease.

From a clinical perspective, these results highlight the relevance of incorporating psychoemotional assessment into the routine management of hemodialysis patients, using nutritional and metabolic parameters as complementary indicators of potential emotional vulnerability. Identifying these patterns may support more personalized psychosocial and nutritional approaches within nephrology practice.

Future research should validate these findings in larger, multicenter, and longitudinal cohorts, incorporating variables such as quality of life, treatment adherence, and social support. Such approaches will be essential for clarifying temporal relationships and better understanding the potential directional links between metabolic status and mental health in chronic kidney disease.

## Figures and Tables

**Table 1:** Biochemical parameters, analytical methods, and clinical relevance.

Parameter	Analytical Method/Instrument	Units	Reference Range	Clinical Relevance
β_2_-microglobulin	Immunonephelometry (BN II; Siemens Healthcare Diagnostics, Erlangen, Germany)	mg/dL	1.0–3.0	Marker of middle-molecule clearance, inflammation, and dialysis biocompatibility.
Albumin	Colorimetry with bromocresol green (Cobas 8000; Roche Diagnostics, Basel, Switzerland)	g/dL	3.5–5.0	Reflects nutritional and inflammatory status; hypoalbuminemia predicts morbidity.
Hemoglobin	Automated spectrophotometric hematology analysis (XN-1000; Sysmex Corporation, Kobe, Japan)	g/dL	12–16 (M); 11–15 (F)	Indicator of erythropoietic function and anemia control.
Hematocrit	Derived from red blood cell indices (XN-1000; Sysmex Corporation, Kobe, Japan)	%	36–46 (M); 33–43 (F)	Correlates with oxygen-carrying capacity.
Phosphorus	UV colorimetry with molybdate (Cobas 8000; Roche Diagnostics, Basel, Switzerland)	mg/dL	2.5–4.5	Marker of mineral-bone metabolism; hyperphosphatemia linked to vascular calcification.
Potassium	Ion-selective electrode potentiometry (Cobas 8000; Roche Diagnostics, Basel, Switzerland)	mEq/L	3.5–5.5	Reflects electrolyte balance; abnormalities may influence anxiety and neuromuscular excitability.
Iron	Colorimetry with ferrozine (Cobas 8000; Roche Diagnostics, Basel, Switzerland)	µg/dL	60–160	Indicator of iron stores and erythropoiesis; deficiency associated with fatigue and low mood.
Calcium	Colorimetry with arsenazo III (Cobas 8000; Roche Diagnostics, Basel, Switzerland)	mg/dL	8.5–10.2	Related to neuromuscular excitability and bone mineralization.
25-hydroxyvitamin D [25(OH)D]	Chemiluminescent immunoassay (Architect i2000SR; Abbott Diagnostics, Abbott Park, IL, USA)	ng/mL	30–100	Low levels associated with inflammation, fatigue, and depressive symptoms.

Note. All biochemical variables were expressed as continuous quantitative measures. Variables showing positively skewed distributions (β_2_-microglobulin, phosphorus, and 25(OH)D) were log-transformed [ln(x)] prior to parametric statistical analyses.

**Table 2:** Psychological variables (dependent outcomes).

Variable	Statistical Type	Scale	Notes
Depression (DASS-21)	Quasi-Continuous (treated as quantitative)	Interval	Sum of 7 items (range 0–42); originally ordinal but analyzed as continuous due to distributional properties.
Anxiety (DASS-21)	Continuous	Interval	Same structure and analytic treatment as Depression.
Stress (DASS-21)	Continuous	Interval	Same as above.

Note. Although technically ordinal, DASS-21 subscale scores are conventionally analyzed as quasi-continuous when normality assumptions or robust tests (e.g., Welch’s ANOVA, Spearman’s *ρ*) are applied.

**Table 3:** Biochemical parameters (independent variables).

Variable	Statistical Type	Scale	Units/Method	Notes
β_2_-microglobulin	Continuous	Ratio	mg/dL; immunonephelometry	Log-transformed when non-normal; marker of inflammation and middle-molecule clearance.
Albumin	Continuous	Ratio	g/dL; colorimetry	Normally distributed; reflects nutritional and inflammatory status.
Hemoglobin	Continuous	Ratio	g/dL	Marker of anemia and oxygen transport.
Hematocrit	Continuous	Ratio	%	Indicates oxygen-carrying capacity.
Phosphorus	Continuous	Ratio	mg/dL	Log-normalized if skewed; marker of mineral metabolism.
Potassium	Continuous	Ratio	mEq/L	Reflects electrolyte balance; relevant to neuromuscular activity.
Iron	Continuous	Ratio	µg/dL	Reflects iron stores; deficiency associated with fatigue and cognition.
Calcium	Continuous	Ratio	mg/dL	Related to bone and neuromuscular health.
25-hydroxyvitamin D [25(OH)D]	Continuous	Ratio	ng/mL; chemiluminescent immunoassay	Log-transformed if skewed; associated with inflammatory and psychological status.

Note. All biochemical variables were analyzed as continuous measures. Variables with positively skewed distributions (β_2_-microglobulin, phosphorus, and 25[OH]D) were log-transformed [ln(x)] prior to parametric analyses.

**Table 4:** Sociodemographic and clinical variables.

Variable	Statistical Type	Scale	Coding/Description	Notes
Age	Continuous	Ratio	Years	Quantitative covariate.
Gender	Discrete (binary)	Nominal	0 = Men; 1 = Women	Main stratification variable.
Dialysis vintage	Continuous (or categorical)	Ratio/Ordinal	Months; optionally grouped as 1–3, 4–6, >6 years	Used as continuous or categorized variable.
Diabetes mellitus	Discrete	Nominal	0 = No; 1 = Yes	Primary comorbidity.
Cardiovascular disease	Discrete	Nominal	0 = No; 1 = Yes	Secondary comorbidity.

Note. Binary clinical variables were dummy-coded (0 = absence; 1 = presence). Dialysis vintage was analyzed both as a continuous and, when required, as a categorical variable for descriptive purposes.

**Table 5:** Biochemical parameters and dialysis adequacy by gender.

Parameter	Total (*n* = 54)	Men (*n* = 34)	Women (*n* = 20)	Welch’s *p*-Value	Cohen’s *d*
Albumin (g/dL)	3.33 ± 0.93	3.52 ± 1.18	3.02 ± 0.45	0.028	+0.57
β_2_-microglobulin (mg/dL)	2.49 ± 0.80	2.33 ± 0.84	2.73 ± 0.72	0.067	−0.51
Potassium (mEq/L)	4.87 ± 0.72	4.83 ± 0.83	4.93 ± 0.52	0.580	−0.15
Iron (µg/dL)	77.46 ± 41.17	83.60 ± 50.75	67.17 ± 20.04	0.095	+0.43
Calcium (mg/dL)	8.93 ± 0.70	9.13 ± 0.54	8.63 ± 0.82	0.021	+0.72
Vitamin D (ng/mL)	24.60 ± 13.80	28.11 ± 15.74	18.64 ± 8.82	0.006	+0.74

**Note.** Values are expressed as mean ± standard deviation. Comparisons between genders were performed using Welch’s *t*-test for unequal variances. Positive *d* values indicate higher means in men.

**Table 6:** Psychoaffective variables (DASS-21) by gender.

Variable	Total (*n* = 54)	Men (*n* = 34)	Women (*n* = 20)	Welch’s *p*-Value	Cohen’s *d*
Depression (0–3)	0.52 ± 0.51	0.40 ± 0.50	0.70 ± 0.47	0.031	−0.62
Anxiety (0–3)	0.55 ± 0.50	0.43 ± 0.50	0.75 ± 0.44	0.018	−0.68
Stress (0–3)	0.54 ± 0.59	0.37 ± 0.49	0.80 ± 0.70	0.021	−0.71

**Note.** DASS-21 scale: 0 = absent, 1 = mild, 2 = moderate, 3 = severe. Comparisons were performed using Welch’s *t*-test for unequal variances. Negative *d* values indicate higher mean scores in women. Results were consistent with Mann–Whitney tests (*p* < 0.05 for all domains).

**Table 7:** Robust Multiple Linear Regression (HC3) Model for Depression (DASS-21).

Variable	Unstandardized β	*p*-Value	95% CI Lower	95% CI Upper
Albumin	0.074	0.602	−0.103	0.178
Gender	2.305	0.001	0.960	3.831
Albumin × Gender	−1.093	0.126	−0.845	0.104
β_2_-microglobulin	0.288	0.135	−0.056	0.413
β_2_-microglobulin × Gender	−1.057	0.016	−0.695	−0.072
Calcium	−0.319	0.004	−0.389	−0.075
Vitamin D	0.312	0.016	0.002	0.020

Adjusted R^2^ = 0.30 Note. Coefficients correspond to unstandardized β values; confidence intervals are expressed in the original measurement units. Robust multiple linear regression (HC3 estimator). Bold coefficients indicate statistically significant associations (*p* < 0.05). Interaction terms reflect statistical moderation of associations by gender.

**Table 8:** Robust Multiple Linear Regression (HC3) Model for Anxiety (DASS-21).

Variable	Standardized β	*p*-Value	95% CI Lower	95% CI Upper
Albumin	0.092	0.741	−0.229	0.322
Gender	1.941	0.030	0.190	3.827
Albumin × Gender	−0.664	0.468	−0.829	0.381
β_2_-microglobulin	0.153	0.444	−0.147	0.335
β_2_-microglobulin × Gender	−1.091	0.029	−0.748	−0.040
Calcium	−0.268	0.017	−0.355	−0.034
Vitamin D	0.153	0.315	−0.005	0.016

Adjusted R^2^ = 0.15 Note. Robust multiple regression (HC3) including biochemical variables and gender. Significant coefficients (*p* < 0.05) are shown in bold.

**Table 9:** Robust Multiple Linear Regression (HC3) Model for Stress (DASS-21).

Variable	Standardized β	*p*-Value	95% CI Lower	95% CI Upper
Albumin	−0.140	0.056	−0.171	0.002
Gender	1.912	0.063	−0.127	4.887
Albumin × Gender	−1.457	0.208	−1.512	0.330
β_2_-microglobulin	0.039	0.828	−0.231	0.289
β_2_-microglobulin × Gender	−0.092	0.882	−0.569	0.489
Calcium	0.078	0.596	−0.183	0.318
Vitamin D	0.180	0.153	−0.003	0.018

Adjusted R^2^ = 0.12 Note. HC3 robust regression model in which no variables reached conventional statistical significance; albumin showed a marginal association (*p* = 0.056).

**Table 10:** Comparative Summary of Alternative Regression Models (Model Fit and Parsimony Criteria).

Domain	Model	Adjusted R^2^	AIC	BIC	ΔR^2^aj vs. M1
Depression	M1: No interactions	0.22	141.3	158.9	—
	M2: Gender × Albumin	0.26	137.8	157.6	+0.04
	M3: Gender × Albumin + Gender × β_2_-microglobulin	0.30	133.5	155.7	+0.08
Anxiety	M1: No interactions	0.11	145.4	163.5	—
	M2: Gender × Albumin	0.13	143.2	162.9	+0.02
	M3: Gender × Albumin + Gender × β_2_-microglobulin	0.15	139.7	160.8	+0.04
Stress	M1: No interactions	0.10	149.9	168.5	—
	M2: Gender × Albumin	0.11	148.6	169.4	+0.01
	M3: Gender × Albumin + Gender × β_2_-microglobulin	0.12	146.1	168.8	+0.02

Note. Models were fitted using robust multiple linear regression (HC3 estimator). Parsimony was evaluated using the Akaike (AIC) and Bayesian (BIC) information criteria, with where lower values indicating better relative fit. Model M3, which includes both interaction terms (Gender × Albumin and Gender × β_2_-microglobulin), showed the most favorable balance between explained variance and model simplicity across all DASS-21 domains.

**Table 11:** Robustness and Stability Diagnostics of Multivariate Models.

DASS-21 Domain	Cook’s D (Max.)	Influential Cases	Observed Range	DFBETAs (Max. |β|)	Bootstrap Δβ (%)	95% CI Concordance
Depression	0.12	None	0.00–0.12	0.83	3.4	100%
Anxiety	0.09	None	0.00–0.09	0.71	4.8	100%
Stress	0.14	None	0.00–0.14	0.92	2.9	98%

Note. Cook’s D < 1/n (≈0.018) indicates no relevant influence; all observed values were well below this threshold. Δβ (%) represents the relative difference between the original coefficients and those obtained via bootstrap resampling. 95% CI concordance denotes the proportion of parameters that retained identical direction and statistical significance after resampling.

**Table 12:** Summary of Standardized Loadings and Structural Effects (PLS-SEM).

Structural Path	Standardized β	95% CI	*p* (Bootstrap)	Significant
Albumin → GPD	−0.28	[−0.45, −0.09]	0.008	Yes
Calcium → GPD	−0.24	[−0.41, −0.05]	0.015	Yes
β_2_-microglobulin → GPD	+0.34	[0.12, 0.55]	0.006	Yes
Gender → GPD	+0.29	[0.08, 0.49]	0.010	Yes
Vitamin D → GPD	−0.11	[−0.31, 0.07]	0.210	No
Gender × β_2_-microglobulin → GPD	+0.18	[0.02, 0.35]	0.037	Yes

Note. PLS-SEM model summarizing the statistical relationships between biochemical parameters and Global Psychological Distress (GPD). Negative β coefficients indicate inverse associations (higher biochemical values correspond to lower distress), whereas positive β coefficients indicate direct associations (higher biochemical values correspond to higher distress). Gender functions as a statistical moderator, particularly in its interaction with β_2_-microglobulin, reinforcing a differential pattern of emotional vulnerability. The model demonstrated good convergent validity and internal consistency, accounting for a substantial proportion of variance in GPD.

**Table 13:** Biopsychosocial Profiles Identified through Mixed Cluster Analysis.

Profile	Predominant Biochemical Characteristics	Emotional Profile (DASS-21)	Clinical Interpretation
Stable Metabolic	Parameters within optimal ranges; good nutrition and low β_2_-microglobulin	Low depression, anxiety, and stress	Clinically stable and emotionally compensated patients
Catabolic-Emotional	Low albumin and calcium, elevated β_2_-microglobulin, reduced vitamin D	High depression and anxiety	Metabolic vulnerability associated with emotional distress
Compensated Resilient	Mild metabolic alterations.	Minimal emotional distress, good adaptation	Effective coping profile despite physiological imbalance

**Table 14:** Comparative Results of Ridge and LASSO Penalized Models by DASS-21 Domain.

DASS-21 Domain	Variables Retained in Ridge	Variables Retained in LASSO	Statistical Interpretation
Depression	β_2_-microglobulin (+), Calcium (−), Albumin (−), Gender (+)	β_2_-microglobulin (+), Calcium (−), Gender (+)	LASSO confirms the weight of the most consistent variables and removes minor redundancies
Anxiety	β_2_-microglobulin (+), Calcium (−), Albumin (−), Gender (+)	β_2_-microglobulin (+), Calcium (−)	Anxiety aligns with the catabolic profile (high β_2_-microglobulin, low calcium) rather than nutritional variables
Stress	Albumin (−), Calcium (−), β_2_-microglobulin (+)	Albumin (−), Calcium (−)	Stress is linked to metabolic–protein equilibrium rather than gender differences

**Table 15:** Sensitivity and Robustness Analyses of Multivariate Models.

Procedure	Variables Evaluated	Model Type	Relative Change in β (%)	Significant Predictors Preserved	Directional Concordance (%)
Log transformation (β_2_-microglobulin, Vitamin D)	β_2_-microglobulin, Vitamin D	Robust linear (HC3)	<5%	Yes	100
Outlier exclusion (z > 3)	All biomarkers	Robust linear (HC3)	<5%	Yes	98
Theil–Sen regression	All biomarkers	Nonparametric	<7%	Yes	96
Quadratic terms	Albumin, Calcium, β_2_-microglobulin	Linear with polynomial terms	<3%	No new ones	100
Bootstrap (5000 iterations)	β_2_-microglobulin, Albumin, Calcium	Standardized biochemical model	—	Yes	100

Note. Directional concordance indicates the percentage of predictors maintaining the same coefficient sign and significance (*p* < 0.05) relative to the baseline model.

## Data Availability

The datasets generated and analyzed during the current study are available from the corresponding author upon reasonable request. Due to privacy restrictions and institutional data protection policies, individual-level patient data cannot be publicly shared. Aggregated and anonymized data supporting the main findings are available in the Supplementary Material. The datasets generated and/or analyzed during the current study are not publicly available due to institutional policies but are available from the corresponding author upon reasonable request (G.M.Z.F.; gzarfer@mde.es). All figures and tables were created by the authors and do not reproduce or adapt content from other sources.

## Supplementary Materials

The following supporting information can be downloaded at: https://www.mdpi.com/article/10.3390/biomedicines13123092/s1, Supplementary File: STROBE Checklist for Cross-Sectional Observational Studies; Table S1: Baseline sociodemographic and clinical characteristics by gender (combined descriptive statistics and effect sizes); Table S2: Spearman Correlations Between DASS-21 Subscales and Biochemical Parameters; Table S3: Spearman Correlations Between DASS-21 Scores and Biochemical Parameters in Men; Table S4: Spearman Correlations Between DASS-21 Scores and Biochemical Parameters in Women; Table S5: Integrative Summary of Multivariate Predictors of Depression, Anxiety, and Stress; Table S6: Cross-Validation Results of Predictive Models (LOOCV and k-fold); Table S7: Post-hoc Statistical Power and Global Effect Size of Multivariate Models.

## Abbreviations

AIC	Akaike Information Criterion
ANOVA	Analysis of Variance
BCa	Bias-Corrected and Accelerated Bootstrap
BIC	Bayesian Information Criterion
CI	Confidence Interval
CKD	Chronic Kidney Disease
DASS-21	Depression, Anxiety and Stress Scale—21 items
df	Degrees of Freedom
DFBETAs	Difference in Beta Statistics
FDR	False Discovery Rate
HD	Hemodialysis
HC3	Heteroscedasticity-Consistent Covariance Matrix Estimator, Type 3
IQR	Interquartile Range
LASSO	Least Absolute Shrinkage and Selection Operator
MANOVA	Multivariate Analysis of Variance
OR	Odds Ratio
PLS-SEM	Partial Least Squares Structural Equation Modeling
q	Adjusted *p*-value after FDR correction (Benjamini–Hochberg)
R	Spearman’s Correlation Coefficient (rho)
R^2^	Coefficient of Determination
R^2^aj	Adjusted Coefficient of Determination
SD	Standard Deviation
SE	Standard Error
SPSS	Statistical Package for the Social Sciences
STROBE	Strengthening the Reporting of Observational Studies in Epidemiology
VIF	Variance Inflation Factor
25(OH)D	25-hydroxyvitamin D
