# Supplementary material for: Biochemical Associations with Depression, Anxiety, and Stress in Hemodialysis: The Role of Albumin, Calcium, and β2-Microglobulin According to Gender
Source: Biomedicines. 2025 Dec 15;13(12):3092. doi: 10.3390/biomedicines13123092 (PMC12731038; doi:10.3390/biomedicines13123092)
Supplement: Supplementary file 1 [file biomedicines-13-03092-s001.zip › Supplementary Table S1.pdf]

Table S1. Baseline sociodemographic and clinical characteristics by gender (combined descriptive statistics and effect sizes).

Variable	Total (*n* = 54)	Men (*n* = 34)	Women (*n* = 20)	Effect size (95 % CI)	*p*-value[ab]
**Age (years)**	67.8 ± 14.9	66.0 ± 15.8	70.9 ± 13.4	Hedges' g = –0.33 [–0.87, +0.21]	.238[a]
**Time on HD (years)**	2.02 ± 0.77	2.05 ± 0.81	1.98 ± 0.73	Hedges' g = +0.09 [–0.44, +0.61]	.738[a]
**Session duration (min)**	232.2 ± 18.5	233.6 ± 17.3	230.0 ± 20.2	Hedges' g = +0.18 [–0.35, +0.71]	.511[a]
**Marital status**, *n* (%)				OR = 1.12 [0.43, 2.92]; RD = +4.5 pp [–17.2, +25.9]	.81[b]
├── Single	15 (27.8)	9 (26.5)	6 (30.0)		
├── Married	27 (50.0)	17 (50.0)	10 (50.0)		
├── Widowed	8 (14.8)	5 (14.7)	3 (15.0)		
└── Divorced	4 (7.4)	3 (8.8)	1 (5.0)		
**Educational level**, *n* (%)				OR = 0.89 [0.35, 2.28]; RD = –3.7 pp [–24.5, +17.6]	.77[b]
├── No formal education	4 (7.4)	2 (5.9)	2 (10.0)		
├── Primary education	17 (31.5)	10 (29.4)	7 (35.0)		
├── Secondary education	19 (35.2)	13 (38.2)	6 (30.0)		
├── Vocational training	8 (14.8)	5 (14.7)	3 (15.0)		
└── University degree	6 (11.1)	4 (11.8)	2 (10.0)		
**Diabetes mellitus**, *n* (%)	18 (33.3)	11 (32.4)	7 (35.0)	OR = 0.89 [0.30, 2.66]; RD = –2.6 pp [–24.4, +19.2]	.84[b]
**Cardiovascular disease**, *n* (%)	20 (37.0)	12 (35.3)	8 (40.0)	OR = 0.82 [0.28, 2.40]; RD = –4.7 pp [–27.4, +18.1]	.74[b]

Notes. *Continuous variables are expressed as mean ± SD; categorical variables as n (%). Positive Hedges' g indicates higher mean in men. ORs (men* vs. *women) and RDs are given with 95 % CIs.* [a]*Welch's t-test (unequal variances).* [b]*Fisher's exact test (two-tailed). Effect sizes with 95% CIs complement p-values. Abbreviations:* ***HD****: hemodialysis;* ***OR****: odds ratio;* ***RD****: risk difference;* ***CI*** *: confidence interval;* ***SD****: standard deviation.*
