# Supplementary material for: Biochemical Associations with Depression, Anxiety, and Stress in Hemodialysis: The Role of Albumin, Calcium, and β2-Microglobulin According to Gender
Source: Biomedicines. 2025 Dec 15;13(12):3092. doi: 10.3390/biomedicines13123092 (PMC12731038; doi:10.3390/biomedicines13123092)
Supplement: Supplementary file 1 [file biomedicines-13-03092-s001.zip › Supplementary Table S2.pdf]

Table S2. Spearman Correlations Between DASS-21 Subscales and Biochemical Parameters.

DASS-21 Domain	Biomarker	Spearman's ρ	p-value	q (FDR)
**Depression**	**Calcium**	**−0.326**	**0.015**	**0.320**
	**β_2-microglobulin**	**+0.284**	**0.035**	**0.248**
	Albumin	−0.260	0.055	0.231
	Iron	−0.152	0.280	0.442
	Potassium	−0.118	0.390	0.481
	Vitamin D	−0.183	0.190	0.372
**Anxiety**	**Calcium**	**−0.289**	**0.032**	**0.340**
	Albumin	−0.257	0.059	0.205
	β_2-microglobulin	+0.208	0.140	0.301
	Iron	−0.097	0.470	0.493
	Potassium	−0.083	0.530	0.502
	Vitamin D	−0.171	0.220	0.366
**Stress**	**Albumin**	**−0.280**	**0.038**	**0.201**
	β_2-microglobulin	+0.167	0.230	0.361
	Calcium	−0.148	0.300	0.411
	Iron	−0.079	0.550	0.506
	Potassium	−0.071	0.590	0.509
	Vitamin D	−0.139	0.330	0.416

Note. *Spearman's rank-order correlations (two-tailed). The Benjamini–Hochberg false discovery rate (FDR, $q = 0.10$) was applied to control type I error. Negative ρ values indicate inverse associations (lower biomarker levels correspond to higher emotional distress). Values in* ***bold*** *denote $p < 0.05$ before FDR correction.*
