# Supplementary material for: Biochemical Associations with Depression, Anxiety, and Stress in Hemodialysis: The Role of Albumin, Calcium, and β2-Microglobulin According to Gender
Source: Biomedicines. 2025 Dec 15;13(12):3092. doi: 10.3390/biomedicines13123092 (PMC12731038; doi:10.3390/biomedicines13123092)
Supplement: Supplementary file 1 [file biomedicines-13-03092-s001.zip › Supplementary Table S3.pdf]

Table S3. Spearman Correlations Between DASS-21 Scores and Biochemical Parameters in Men.

DASS-21 Domain	Biomarker	Spearman's ϱ	p-value	q (FDR)
**Depression**	Calcium	−0.358	0.037	0.270
	Albumin	−0.322	0.049	0.285
	β_2-microglobulin	+0.267	0.131	0.386
	Iron	−0.155	0.380	0.531
	Potassium	−0.124	0.472	0.544
	Vitamin D	−0.179	0.301	0.486
**Anxiety**	Albumin	−0.334	0.041	0.280
	Calcium	−0.293	0.076	0.315
	β_2-microglobulin	+0.192	0.259	0.471
	Iron	−0.140	0.430	0.536
	Potassium	−0.122	0.477	0.544
	Vitamin D	−0.164	0.352	0.510
**Stress**	Albumin	−0.314	0.049	0.285
	Calcium	−0.276	0.094	0.324
	β_2-microglobulin	+0.205	0.236	0.463
	Iron	−0.163	0.350	0.510
	Potassium	−0.089	0.598	0.586
	Vitamin D	−0.132	0.444	0.537

Note. *Spearman's rank-order correlations (two-tailed). Benjamini–Hochberg FDR correction (q = 0.10) was applied to control type I error. Values in* ***bold*** *indicate nominal statistical significance (p < 0.05). Negative ρ values denote inverse relationships (lower biomarker levels associated with higher emotional distress).*
