# Supplementary material for: Biochemical Associations with Depression, Anxiety, and Stress in Hemodialysis: The Role of Albumin, Calcium, and β2-Microglobulin According to Gender
Source: Biomedicines. 2025 Dec 15;13(12):3092. doi: 10.3390/biomedicines13123092 (PMC12731038; doi:10.3390/biomedicines13123092)
Supplement: Supplementary file 1 [file biomedicines-13-03092-s001.zip › Supplementary Table S4.pdf]

Table S4. Spearman Correlations Between DASS-21 Scores and Biochemical Parameters in Women.

DASS-21 Domain	Biomarker	Spearman's ρ	p-value	q (FDR)
**Depression**	β_2-microglobulin	+0.447	0.045	0.285
	Albumin	−0.304	0.180	0.415
	Calcium	−0.282	0.212	0.428
	Iron	−0.229	0.311	0.453
	Potassium	−0.194	0.382	0.482
	Vitamin D	−0.250	0.268	0.441
**Anxiety**	Albumin	−0.268	0.236	0.434
	β_2-microglobulin	+0.361	0.118	0.386
	Calcium	−0.219	0.329	0.453
	Iron	−0.204	0.358	0.468
	Potassium	−0.156	0.476	0.528
	Vitamin D	−0.198	0.370	0.470
**Stress**	β_2-microglobulin	+0.324	0.157	0.398
	Albumin	−0.271	0.230	0.431
	Calcium	−0.241	0.285	0.444
	Iron	−0.188	0.403	0.477
	Potassium	−0.167	0.456	0.523
	Vitamin D	−0.222	0.324	0.452

Note. *Spearman's rank-order correlations (two-tailed). Benjamini–Hochberg FDR correction ($q = 0.10$) applied to control for multiple comparisons. Values in bold indicate nominal significance ($p < 0.05$). Positive ρ values represent direct relationships (higher biomarker levels associated with higher emotional distress).*
