# Supplementary material for: Biochemical Associations with Depression, Anxiety, and Stress in Hemodialysis: The Role of Albumin, Calcium, and β2-Microglobulin According to Gender
Source: Biomedicines. 2025 Dec 15;13(12):3092. doi: 10.3390/biomedicines13123092 (PMC12731038; doi:10.3390/biomedicines13123092)
Supplement: Supplementary file 1 [file biomedicines-13-03092-s001.zip › Supplementary Table S5.pdf]

Table S5. Integrative Summary of Multivariate Predictors of Depression, Anxiety, and Stress.

Predictor	Associated Affective Domain	Direction of Effect	Significance in Robust/Penalized Models	Evidence of Gender Interaction	Stability (Bootstrap/Log-transform)
Albumin (g/dL)	Depression, Anxiety, Stress	Negative	High / Moderate	Mild moderator (Gender × Albumin)	High (consistent across all models)
β_2-microglobulin (mg/dL)	Depression > Anxiety	Positive	High	Significant moderator (Gender × β_2-microglobulin)	High (robust after log-transformation)
Calcium (mg/dL)	Depression, Anxiety	Negative	High	Not significant	High
Vitamin D (ng/mL)	Depression (trend)	Slightly positive	Marginal	Not significant	Moderate
Gender (Female)	Depression, Anxiety, Stress	Positive	High	—	High
Age, Comorbidities (Diabetes, CVD)	All	Neutral / Weak	Not significant	—	High (no influence)

Note. *Direction of effect indicates the relationship between the predictor and emotional severity (negative = protective; positive = vulnerability effect). Significance columns integrate results from robust (HC3), ridge, and LASSO models. Stability was evaluated* via *5,000 bootstrap iterations and log-transformations for asymmetric variables.*
