# Supplementary material for: Biochemical Associations with Depression, Anxiety, and Stress in Hemodialysis: The Role of Albumin, Calcium, and β2-Microglobulin According to Gender
Source: Biomedicines. 2025 Dec 15;13(12):3092. doi: 10.3390/biomedicines13123092 (PMC12731038; doi:10.3390/biomedicines13123092)
Supplement: Supplementary file 1 [file biomedicines-13-03092-s001.zip › Supplementary Table S6.pdf]

Table S6. Cross-Validation Results of Predictive Models (LOOCV and k-fold).

DASS-21 Domain	Model Type	Validation Method	Cross-Validated MSE (ECMcv)	r_pred (Predicted–Observed)	Relative Difference vs. Original (%)	Interpretation
**Depression**	HC3 robust	LOOCV	Low	High	< 5	High stability and predictive precision
	Ridge	k = 10	Low	High	< 5	Penalization enhances parsimony
	LASSO	k = 10	Low	High	< 7	Simplification without loss of fit
**Anxiety**	HC3 robust	LOOCV	Low–medium	Moderate–high	< 10	Adequate stability
	Ridge	k = 10	Low	Moderate–high	< 8	Stable model
	LASSO	k = 10	Low	Moderate	< 10	Slight complexity reduction
**Stress**	HC3 robust	LOOCV	Medium	Moderate	< 10	Acceptable stability
	Ridge	k = 10	Medium	Moderate	< 9	Well-adjusted model
	LASSO	k = 10	Medium	Moderate–low	< 10	Slight fit reduction, higher parsimony

Note. ECMcv*: mean squared error under cross-validation;* ***r_pred****: correlation between predicted and observed values. Relative difference refers to the percentage variation in error compared with the non-validated model. The robust (HC3) and penalized (Ridge, LASSO) models yielded comparable predictive performance and no evidence of overfitting, confirming overall reliability and generalizability.*
