# Supplementary material for: Biochemical Associations with Depression, Anxiety, and Stress in Hemodialysis: The Role of Albumin, Calcium, and β2-Microglobulin According to Gender
Source: Biomedicines. 2025 Dec 15;13(12):3092. doi: 10.3390/biomedicines13123092 (PMC12731038; doi:10.3390/biomedicines13123092)
Supplement: Supplementary file 1 [file biomedicines-13-03092-s001.zip › Supplementary Table S7.pdf]

Table S7. Post-hoc Statistical Power and Global Effect Size of Multivariate Models.

DASS-21 Domain	Model Type	Adjusted R^2	Estimated f^2	Power (1–β)	Effect Size Interpretation	Conclusion
**Depression**	HC3 robust	Moderate–high	0.30–0.35	> 0.90	Large effect	Excellent power; robust findings
	Ridge	Moderate	0.25–0.30	≈ 0.88	Moderate–large	Stable performance
	LASSO	Moderate	0.22–0.28	≈ 0.85	Moderate	Balanced fit–parsimony ratio
**Anxiety**	HC3 robust	Moderate	0.18–0.25	≈ 0.82	Moderate	Adequate statistical power
	Ridge	Moderate	0.17–0.23	≈ 0.80	Moderate	Inter-model consistency
	LASSO	Low–moderate	0.15–0.20	≈ 0.78	Medium	Slightly lower precision
**Stress**	HC3 robust	Low–moderate	0.12–0.18	≈ 0.70	Medium	Acceptable power; possible underdetection
	Ridge	Low–moderate	0.11–0.17	≈ 0.69	Medium	Stable performance
	LASSO	Low	0.10–0.15	≈ 0.65	Small–medium	Expected limitation due to emotional variability

***Note. R^2** :adjusted coefficient of determination; **f^2**: Cohen's effect size; **1–β**:observed statistical power. Effect sizes were computed using the formula $f^2 = R^2 / (1–R^2)$, with simulated estimates based on sample size and number of predictors. The depression and anxiety models exceeded the desirable power threshold (≥ 0.80), indicating adequate inferential capacity.*
