# Supplementary material for: Biochemical Associations with Depression, Anxiety, and Stress in Hemodialysis: The Role of Albumin, Calcium, and β2-Microglobulin According to Gender
Source: Biomedicines. 2025 Dec 15;13(12):3092. doi: 10.3390/biomedicines13123092 (PMC12731038; doi:10.3390/biomedicines13123092)
Supplement: Supplementary file 1 [file biomedicines-13-03092-s001.zip › STROBE_Checklist_Supplementary_.pdf]

Supplementary File STROBE Checklist for Cross-Sectional Observational Studies

Checklist prepared according to the STROBE Statement (von Elm E et al., 2007) for cross-sectional observational studies. The table indicates where each checklist item is addressed in the manuscript entitled ' Biochemical associations with depression, anxiety, and stress in hemodialysis: the role of albumin, calcium, and β_2-microglobulin according to gender'. Locations are specified by section, paragraph, and page numbers for clarity.

Section / Item	STROBE Recommendation	Location in Manuscript (Section / Paragraph / Page)	Compliance
1. Title and Abstract	Indicate study design in title and provide informative summary.	Title and Abstract (Section 1, paragraphs 1–2; pp. 1–2)	Compliant
2. Background/Rationale	Explain scientific background and rationale for investigation.	Introduction (Section 1.1, paragraphs 1–4; pp. 3–5)	Compliant
3. Objectives	State specific objectives and hypotheses.	Introduction (final paragraph; p. 5)	Compliant
4. Study Design	Present key elements of study design early in the paper.	Methods (Section 2.1; paragraph 1; p. 6)	Compliant
5. Setting	Describe setting, locations, and dates of data collection.	Methods (Section 2.1; paragraphs 2–3; pp. 6–7)	Compliant
6. Participants	Provide eligibility criteria and methods of selection.	Methods (Section 2.1; paragraphs 3–4; pp. 6–7)	Compliant
7. Variables	Define outcomes, exposures, predictors, and confounders.	Methods (Sections 2.3.1–2.3.3; paragraphs 1–4; pp. 8–10)	Compliant
8. Data Sources/Measurement	Describe data collection methods for each variable.	Methods (Section 2.3; pp. 8–10)	Compliant
9. Bias	Describe efforts to address potential sources of bias.	Methods (Section 2.4; paragraph 2; pp. 11–12)	Compliant
10. Study Size	Explain how study size was determined.	Methods (Section 2.1; paragraph 5; p. 12)	Compliant
11. Quantitative Variables	Explain how quantitative variables were handled in analyses.	Methods (Section 2.3.3; pp. 12–13)	Compliant
12. Statistical Methods	Describe all statistical methods, including control for confounding and sensitivity analyses.	Methods (Sections 2.4–2.5; paragraphs 1–3; pp. 13–17)	Compliant
13. Participants	Report numbers of individuals at each	Results (Section 3.1; Figure 1; pp. 17–18)	Compliant

	stage of study and reasons for non-participation.		
14. Descriptive Data	Provide characteristics of study participants.	Results (Section 3.1; Table 5; pp. 18–21)	Compliant
15. Outcome Data	Report numbers of outcome events or summary measures.	Results (Sections 3.3–3.4; Tables 7–8; pp. 21–24)	Compliant
16. Main Results	Provide unadjusted and adjusted estimates with precision and confounder control.	Results (Sections 3.6–3.9; Tables 11–14; pp. 24–33)	Compliant
17. Other Analyses	Report subgroup, interaction, or sensitivity analyses.	Results (Sections 3.7–3.12; Figures 8–14; pp. 33–38)	Compliant
18. Key Results	Summarize key findings with reference to objectives.	Discussion (Section 4.1; pp. 38–39)	Compliant
19. Limitations	Discuss limitations, potential bias, and imprecision.	Discussion (Strengths and Limitations subsection; Section 4.3; pp. 39–40)	Compliant
20. Interpretation	Provide interpretation considering objectives, limitations, and literature.	Discussion (Sections 4.4–4.5; pp. 40–42)	Compliant
21. Generalizability	Discuss external validity and contextual transferability.	Discussion (Section 4.5; paragraph 3; p. 42)	Compliant
22. Funding	Give source of funding and role of funders.	Ethics and Funding statement (final paragraph; p. 43)	Compliant

All 22 items were fulfilled according to the official STROBE guidelines for cross-sectional observational studies. The manuscript demonstrates full compliance (22/22).

Supplementary Table S1. STROBE checklist provided as per journal submission guidelines (Biomedicines).
